# Motion correction algorithm improves right coronary artery image quality at heart rates > 75 bpm in non‐ECG‐gated coronary CT angiography

**DOI:** 10.1002/acm2.70803

**Published:** 2026-09-15

**Authors:** Meiqi Wang, Li Tao, Wen Li, YouHong Cao, Hao Wu, Zhiwei Zhang

**Affiliations:** ^1^ Department of Radiology The First Affiliated Hospital of Chongqing Medical University Chongqing China

**Keywords:** artifacts, Coronary computed tomography angiography, heart rate, motion correction

## Abstract

**Background:**

Motion artifacts caused by cardiac pulsation remain a major technical challenge in coronary CT angiography (CCTA). Although an advanced cardiac motion correction (CMC) algorithm has been developed and successfully implemented in clinical practice, the effectiveness of this algorithm in improving coronary image quality in clinical settings has not yet been fully investigated.

**Purpose:**

To evaluate the impact and clinical value of a motion correction algorithm on image quality in non‐ECG‐gated CCTA.

**Methods:**

We retrospectively enrolled 76 patients who underwent CCTA for suspected coronary artery disease. Based on the mean heart rate (HR) recorded during scanning, patients were divided into three groups: low HR (HR ≤ 60 bpm), intermediate HR (60 < HR < 75 bpm), and high HR (HR ≥ 75 bpm). All images were reconstructed using both standard algorithm (STD) and CMC algorithms. The performance of STD and CMC was compared across different HR conditions in terms of subjective (4‐point Likert scale, interpretability) and objective image quality (signal‐to‐noise ratio (SNR), contrast‐to‐noise ratio (CNR), and vessel sharpness).

**Results:**

At high HRs, the difference in subjective image quality of the right coronary artery (RCA) between STD and CMC reached statistical significance (*p* < 0.05). Compared with STD, CMC significantly improved the interpretability of the RCA at high HRs (*p* < 0.001). No statistically significant differences were observed between the two algorithms in terms of SNR or CNR (*p* > 0.05). However, at high HRs, CMC was significantly superior to STD with respect to vessel sharpness of the RCA (*p* < 0.001).

**Conclusions:**

The CMC improves image quality in CCTA, with particularly notable benefits for the RCA under high HR conditions.

## 1 INTRODUCTION

Coronary computed tomography angiography (CCTA) is a noninvasive diagnostic **modality** for coronary artery disease, enabling effective assessment of coronary anatomy as well as hemodynamic parameters.^1^, ^2^, ^3^ Advances in both hardware and software technologies have **substantially** enhanced the clinical value of CCTA.^4^, ^5^ Recent studies have confirmed the clinical feasibility of non‐ECG‐gated techniques in CCTA.^6^ Compared with conventional ECG‐gated CCTA, non‐ECG‐gated CCTA offers advantages when ECG signals are poor, the need for concurrent cardiac monitoring or other interventions during scanning may preclude the attachment of surface ECG electrodes, or circumstances exist in which surface electrode placement is either challenging or contraindicated.^7^, ^8^

However, motion artifacts caused by cardiac pulsation remain a major technical challenge in CCTA. Therefore, effective reduction of these artifacts is essential to ensure diagnostic accuracy. Multiple strategies have been developed to correct motion artifacts, which can be broadly categorized into hardware and software approaches. Hardware strategies include increasing gantry rotation speed to shorten single‐acquisition time, expanding detector coverage to enable whole‐heart capture within a single cardiac cycle, and employing dual‐source CT technology to improve temporal resolution.^9^, ^10^, ^11^ Software approaches include motion correction algorithms and deep learning‐based artificial intelligence techniques.^12^, ^13^

In recent years, an advanced motion‐compensated reconstruction algorithm (CMC, Coronary Motion Clear, **Neusoft** Medical Systems) has been developed and introduced into clinical practice. As a core technology for cardiac CT image reconstruction, this algorithm automatically assesses motion metrics in CCTA images and initiates optimization procedures, thereby enhancing the temporal resolution of cardiac images at the mathematical algorithm level. By optimizing the motion assessment model to align with the actual motion state of the vessels, the algorithm enhances coronary motion compensation and effectively suppresses artifacts caused by suboptimal breath‐holding and cardiac pulsation. The core of the algorithm lies in constructing motion vectors for each pixel to evaluate artifact severity and selecting the scheme with minimal motion artifacts to optimize motion function parameters, thereby dynamically tracking cardiac motion trajectories that cannot be fully resolved by temporal resolution alone.^14^ To our knowledge, the efficacy of this algorithm in improving coronary image quality in clinically relevant settings has not been thoroughly investigated. Therefore, this study aimed to investigate the effect of CMC on image quality in non‐ECG‐gated coronary CT angiography under low, intermediate, and high heart rate (HR) conditions, relative to the standard (STD) algorithm.

## 2 MATERIALS AND METHODS

### 2.1 Study population

**FIGURE 1:**
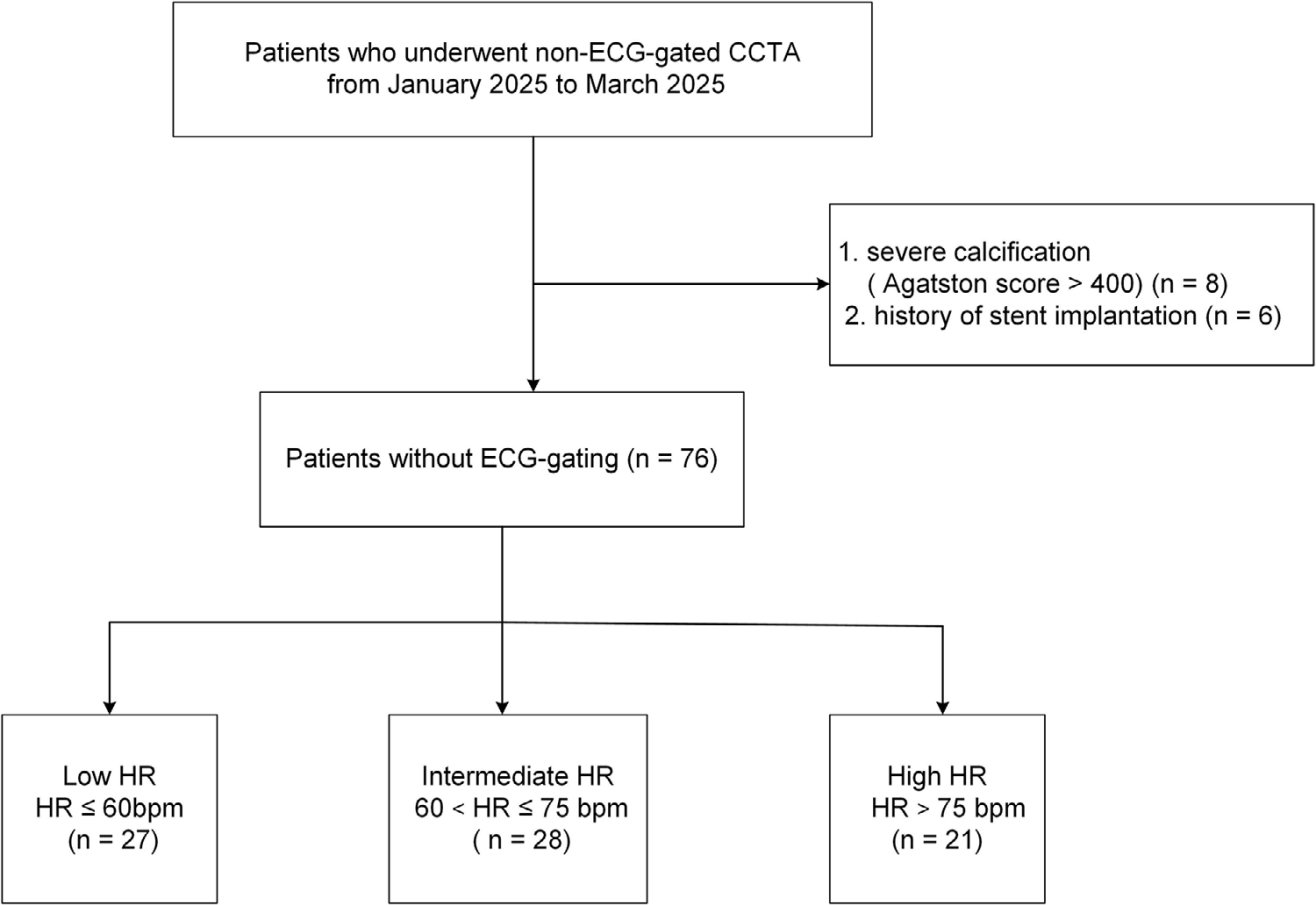
Patient enrollment flowchart. HR: heart rate.

This retrospective study was conducted in accordance with the Declaration of Helsinki and was approved by the Institutional Review Board of our institution, which waived the requirement for individual informed consent (approval number: 2025–298–01). We consecutively enrolled 90 patients who underwent non‐ECG‐gated CCTA at our institution from January 2025 to March 2025. Inclusion criteria were patients referred for CCTA due to chest pain or suspected coronary artery disease. Exclusion criteria were (1) severe coronary calcification (Agatston score > 400) and (2) a history of coronary stent implantation. Enrolled patients were stratified by HR during scanning into three groups: low HR (< 60 bpm), intermediate HR (60–75 bpm), and high HR (> 75 bpm). Figure 1 presents the flowchart of patient enrollment.

### 2.2 Imaging protocol

All CCTA examinations were performed using the axial scanning mode on a 256‐row, 512‐slice CT scanner (NeuViz Epoch, Neusoft Medical Systems, China) equipped with a 16‐cm wide detector. During the examination, real‐time HR was monitored using a finger pulse oximeter (MD300C, Beijing Choice, China), and the mean HR recorded immediately after the CCTA sequence scan was used for patient classification. A coronary calcium scoring scan was performed first, and the scanning range was determined by the size of the heart. A weight‐based contrast injection protocol (Certegra P3T, Bayer Healthcare, Germany) was used.^15^ Iodinated contrast medium (Iopromide, 370 mg I/mL, Bayer Healthcare, Germany) was administered at a flow rate of 3.6–4.8 mL/s, followed by a 40 mL saline flush at the same rate. The region of interest (ROI) was placed in the descending aorta, and scanning was triggered when the real‐time raw CT attenuation value measured by the bolus tracking monitoring scan reached the preset absolute threshold of 150 HU. The acquisition time window was set to 700 ms. Scan parameters were as follows: gantry rotation time 0.259 s, reconstruction thickness 0.625 mm, reconstruction interval 0.3125 mm, tube voltage 100 kV, and automatic tube current modulation applied. None of the patients in this study had taken any medication prior to the examination.

### 2.3 Image reconstruction

After image acquisition, the system automatically recommends four different phase datasets. An experienced radiologist manually selected the phase with optimal image quality for subsequent reconstruction. In the standard reconstruction group, images were reconstructed using the STD algorithm, which is based on ClearView iterative reconstruction technology and incorporates a noise reduction function. In the motion correction group, the CMC algorithm—which builds on STD by adding motion correction capability—was further applied. CMC acquires coronary artery centerlines from the target and adjacent phases through single‐phase multi‐angle acquisition, constructs a voxel‐wise motion vector field, and applies it during back‐projection to generate motion‐corrected images.^16^ The reconstructed images from two groups were then transferred to an dedicated workstation (AVW3.0, Neusoft Medical Systems, China), where the radiologist performed volume rendering, multiplanar reformats, curved planar reformation, and maximum intensity projection.

### 2.4 Subjective image quality assessment

Coronary arteries were divided into 15 segments according to the American Heart Association guidelines.^17^ A 4‐point Likert scale was used: 4 = continuous vessel course, sharp borders, and no motion artifacts; 3 = continuous vessel course, sharp borders, with mild blurring or minor artifacts; 2 = moderately delineated vessel borders with artifacts present, but image quality remains diagnostically acceptable; and 1 = severe artifacts that prevent diagnostic evaluation. Two radiologists (with 4 and 7 years of experience, respectively), blinded to the acquisition and reconstruction protocols, independently assessed image quality. In cases of disagreement, a final consensus was reached by adjudication with a senior radiologist (20 years of experience). Interpretability was defined as a segment score ≥ 2. If a coronary segment was deemed non‐interpretable, the corresponding vessel and patient were also considered non‐interpretable.^18^

### 2.5 Objective image quality assessment

A cardiac imaging radiologist with 4 years of experience performed objective image quality analysis. An ROI was placed in the aortic root, immediately cranial to the left coronary sinus, and the standard deviation of attenuation within this ROI was defined as image noise (in Hounsfield units, HU).^19^, ^20^ Additional ROIs were placed in the proximal segments of the left anterior descending artery (LAD), left circumflex artery (LCX), and right coronary artery (RCA), as well as in the adjacent perivascular adipose tissue (PVAT). CT attenuation (HU) was measured three times on consecutive slices, and the mean value was recorded for analysis, with each ROI drawn as large as possible in a round or oval shape, avoiding calcifications and other anatomical structures.^21^ Signal‐to‐noise ratio (SNR) and contrast‐to‐noise ratio (CNR) were calculated using the following formulas: SNR = HU artery/noise and CNR = (HU artery—HU PVAT)/noise as previously described.^8^

**FIGURE 2:**
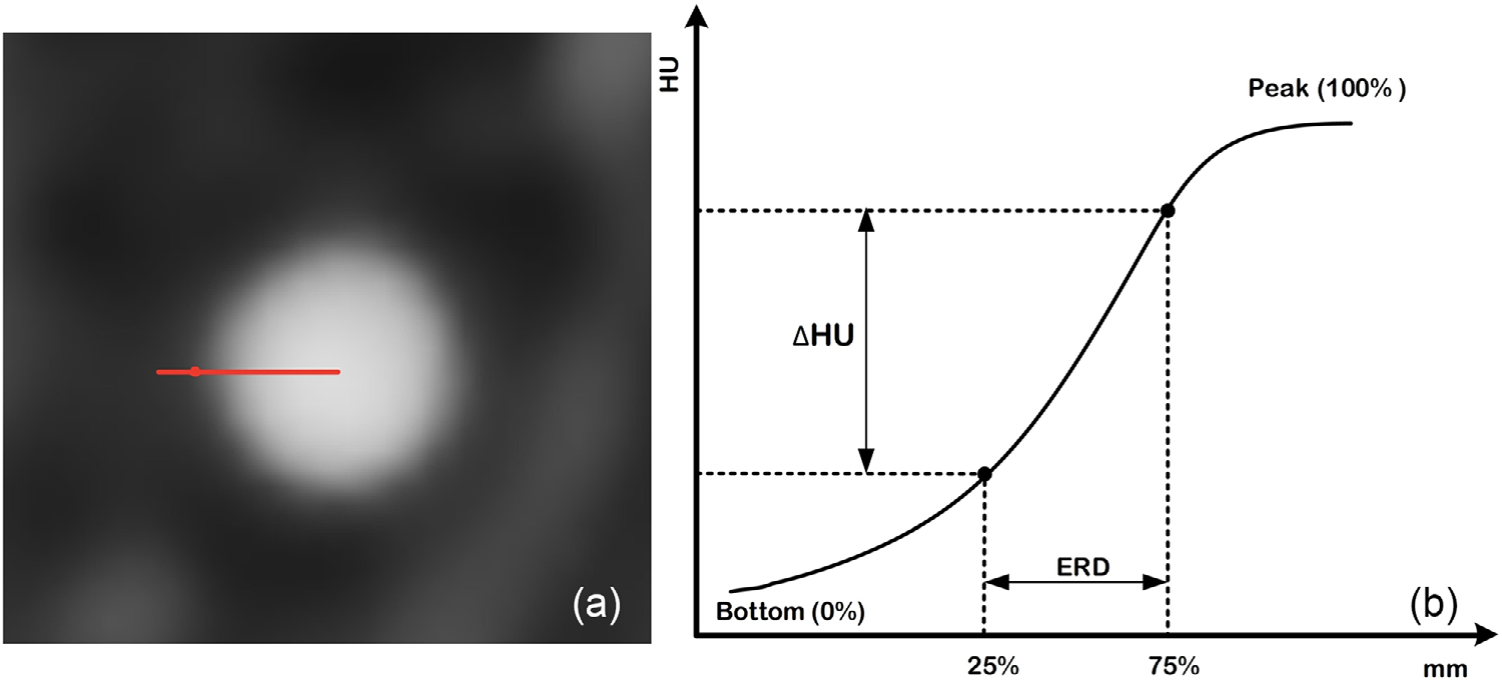
Measurement of vessel sharpness. (a). Profiling a line across the right coronary artery with a reference point placed on the vessel wall. (b). Profile curve showing the 25–75% edge rise distance (ERD) between the wall reference point and peak intensity, with ΔHU derived from the Y‐axis. The edge rise slope (ERS) is calculated as ERS = ΔHU / ERD.

The same radiologist used AVW 1.0 (Neusoft Medical Systems, China) to measure vessel sharpness in the LAD, LCX, and RCA. A measurement line was positioned at the mid‐segment of each of the three major coronary branches, oriented perpendicular to the vessel centerline.^22^ When calcification, plaque, or stenosis was present, measurements were obtained 1.5–2 mm away from the affected area to avoid interference. To minimize partial‐volume effects, only the steepest 25%–75% slope interval was used for evaluation. The edge rise distance (ERD) is defined as the distance between the 25% and 75% points on the CT attenuation curve. Vessel sharpness, or edge rise slope (ERS), is calculated as: (HU75%—HU25%) / ERD (in HU/mm), where HU75% and HU25% denote the CT attenuation values at the 75th and 25th percentiles, respectively. This value represents the maximum slope of the vessel wall attenuation curve and was quantified at the vessel level. Figure 2 illustrates the measurement process for vessel sharpness.

### 2.6 Radiation dose parameters

The dose‐length product (DLP) and volume CT dose index (CTDI_vol_) were recorded for each scan, and the effective radiation dose (ED) was calculated by multiplying the DLP by a chest conversion factor k (k = 0.014 mSv/mGy×cm).^23^

### 2.7 Statistical analysis

Statistical analyses were performed using R software (version 4.4.2). The Shapiro‐Wilk test was used to assess the normality of data distribution. Continuous variables are presented as mean ± standard deviation, and categorical variables as median (Q1, Q3). A patient‐clustered ordinal generalized estimating equation was used to analyze the 4‐point Likert scale scores. A mixed‐effects logistic regression model was applied to assess image interpretability, and a linear mixed‐effects model was used to evaluate vessel sharpness. The Holm‐Bonferroni method was applied for multiple comparison correction. SNR and CNR between the STD and CMC groups were analyzed using the Wilcoxon signed‐rank test. Inter‐observer agreement was evaluated using the kappa coefficient, with the following interpretation: κ < 0.2, poor; 0.21–0.4, fair; 0.41–0.6, moderate; 0.61–0.8, good; and > 0.8, excellent. A *p*‐value < 0.05 was considered statistically significant.

## 3 RESULTS

### 3.1 Patient characteristics

A total of 90 patients who underwent CCTA were initially enrolled in this study. Of these, 8 were excluded due to severe coronary calcification (Agatston score > 400) and 6 were excluded owing to a history of stent implantation, resulting in a final study population of 76 patients. Table 1 summarizes patient characteristics and CT scan parameters.

**TABLE 1:** Patient baseline characteristics.

characteristic	non‐ECG‐gated (*n* = 76)
**Demographic data**	
Age, years ^a^	57.17 ± 10.86
BMI, kg/m^2^ ^a^	24.39 ± 3.17
Average HR bpm ^a^	69.80 ± 11.23
Male/female	39/37
**Cardiovascular risk factors**	
Hypertension, n (%) ^b^	14 (18)
Diabetes, n (%) ^b^	6 (8)
Dyslipidemia, n (%) ^b^	6 (8)
Current smoker, n (%) ^b^	8 (11)
**Radiation dose**	
CTDI_vol_ (mGy) ^a^	13.71 ± 3.24
DLP (mGy) ^a^	193.18 ± 48.04
ED (mSv) ^a^	2.71 ± 0.66

Abbreviations: BMI, body mass index; HR, heart rate; CTDI_vol_, **volume CT dose index**; DLP, dose‐length product; ED, effective dose.

^a^ Data are expressed as mean ± SD.

^b^ Data are expressed as numbers (percentages).

### 3.2 Subjective image quality

**FIGURE 3:**
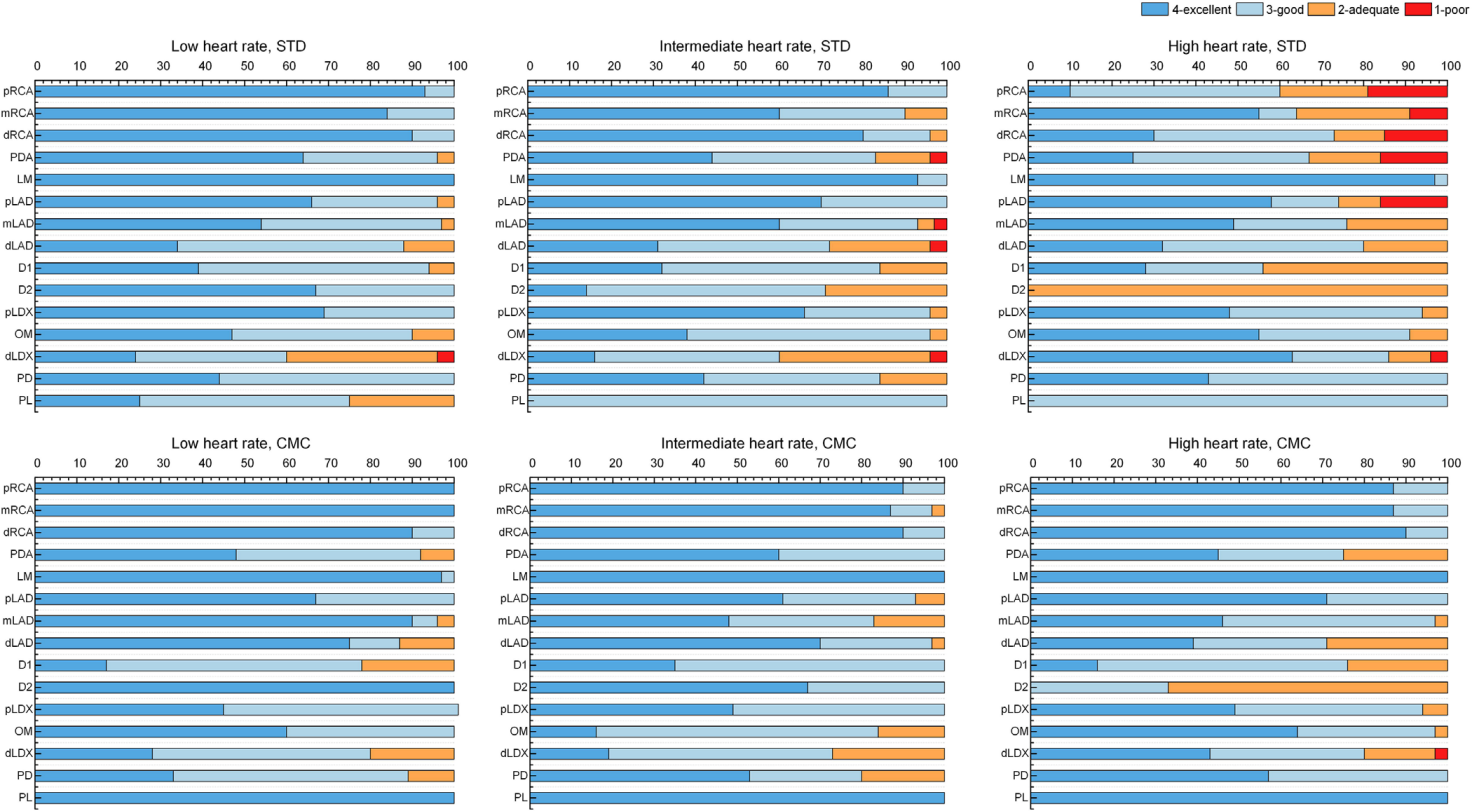
Subjective evaluation of coronary artery segments. The X‐axis (0‐100%) represents the proportion of segments in each group with coronary artery segment scores of 4 (blue), 3 (light blue), 2 (orange), and 1 (red), respectively. The y‐axis represents 15 different coronary artery segments. STD: standard algorithm, CMC: cardiac motion correction. pRCA: proximal right coronary artery, mRCA: mid right coronary artery, dRCA: distal right coronary artery, PDA: posterior descending artery, LM: left main coronary artery, pLAD: proximal left anterior descending artery, mLAD: mid left anterior descending artery, dLAD: distal left anterior descending artery, D1: first diagonal branch of LAD, D2: second diagonal branch of LAD, pLCX: proximal left circumflex artery, OM: obtuse marginal branch of LCX, dLCX: distal left circumflex artery, PD: posterior descending artery, PL: posterolateral branch artery.

Inter‐observer agreement was good (STD: κ = 0.752; CMC: κ = 0.815). As shown in Table 2, at high HRs, the differences in subjective image quality of the RCA between STD and CMC images were statistically significant (*p* < 0.001), whereas no significant differences were observed at other HRs or in other coronary branches. As illustrated in Figure 3 and Table 3, using the STD algorithm, non‐interpretable segments (score = 1) were present in all major coronary artery branches under high HR conditions, with a predominant localization in the RCA. In contrast, with the CMC algorithm, non‐interpretable scores were only present in one non‐major segment.

**TABLE 2:** Subjective image quality scores across different heart rates and algorithms.

	HR ≤ 60 bpm group (*n* = 27)			60 < HR ≤ 75 bpm group (*n* = 28)			HR > 75 bpm group (*n* = 21)		
	RCA	LAD	LCX	RCA	LAD	LCX	RCA	LAD	LCX
STD	4.0(4.0 ‐ 4.0)	4.0(3.0 ‐ 4.0)	3.0(3.0 ‐ 4.0)	4.0(3.0 ‐ 4.0)	4.0(3.0 ‐ 4.0)	3.0(3.0 ‐ 4.0)	3.0(2.0 ‐ 3.0)	3.0(3.0 ‐ 4.0)	4.0(3.0 ‐ 4.0)
CMC	4.0(4.0 ‐ 4.0)	4.0(4.0 ‐ 4.0)	4.0(3.0 ‐ 4.0)	4.0(4.0 ‐ 4.0)	4.0(3.0 ‐ 4.0)	3.0(3.0 ‐ 4.0)	4.0(4.0 ‐ 4.0)	4.0(3.0 ‐ 4.0)	4.0(3.0 ‐ 4.0)
*p*‐value	0.606	0.512	0.606	0.127	0.564	0.564	**< 0.001** ^*^	0.054	0.512

*Note*: Data are presented as median (interquartile range).

Abbreviations: CMC, cardiac motion correction; HR, heart rate, STD, standard algorithm,

* *p*‐value was statistically significant.

**TABLE 3:** Interpretability across different heart rates and algorithms.

	HR ≤ 60 bpm group (*n* = 27)			60 < HR ≤ 75 bpm group (*n* = 28)			HR > 75 bpm group (*n* = 21)		
	STD	CMC	*p*‐value	STD	CMC	*p*‐value	STD	CMC	*p*‐value
Per‐segment									
	99.68 (310/311)	100 (311/311)	0.594	98.80 (329/333)	99.70 (332/333)	0253	92.59 (225/243)	100 (243/243)	**< 0.001** ^*^
Per‐vessel									
LAD	100 (27/27)	100 (27/27)	—	96.43 (27/28)	96.43 (27/28)	0.594	90.48 (18/21)	100 (21/21)	0.253
LCX	96.30 (26/27)	100 (27/27)	0.594	96.43 (27/28)	100 (28/28)	0.594	98.59 (20/21)	100 (21/21)	0.594
RCA	100 (27/27)	100 (27/27)	—	96.43 (27/28)	100 (28/28)	0.594	61.90 (13/21)	100 (21/21)	**< 0.001** ^*^
Per‐patient									
	96.30 (26/27)	100 (27/27)	0.594	88.29 (25/28)	96.43 (27/28)	0.594	57.14 (12/21)	100 (21/21)	**< 0.001** ^*^

*Note*: Interpretability Data are presented as %(n/N).

Abbreviation: CMC, cardiac motion correction; LAD, left anterior descending artery; LCX, left circumflex artery; RCA, right coronary artery; STD, standard algorithm

* *p*‐value was statistically significant.

“‐” indicates statistically non‐comparable measurements between the study groups.

### 3.3 Objective image quality

**FIGURE 4:**
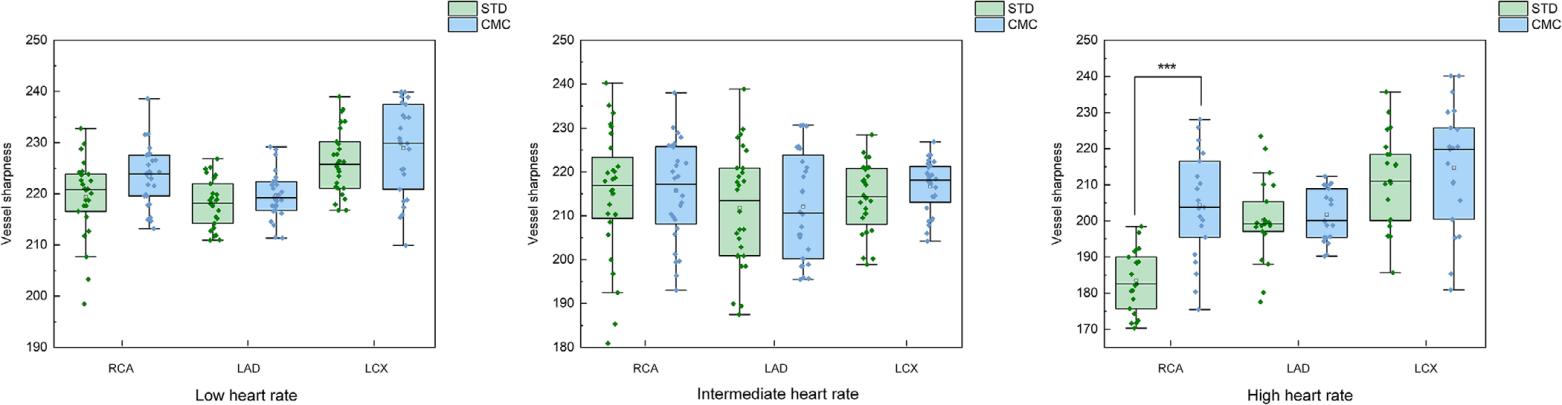
Vessel sharpness measurements for STD and CMC at low, intermediate, and high heart rates. RCA: right coronary artery, LAD: left anterior descending artery, LCX: left circumflex artery, STD: standard algorithm, CMC: cardiac motion correction. ***indicates *p* < 0.001.

**FIGURE 5:**
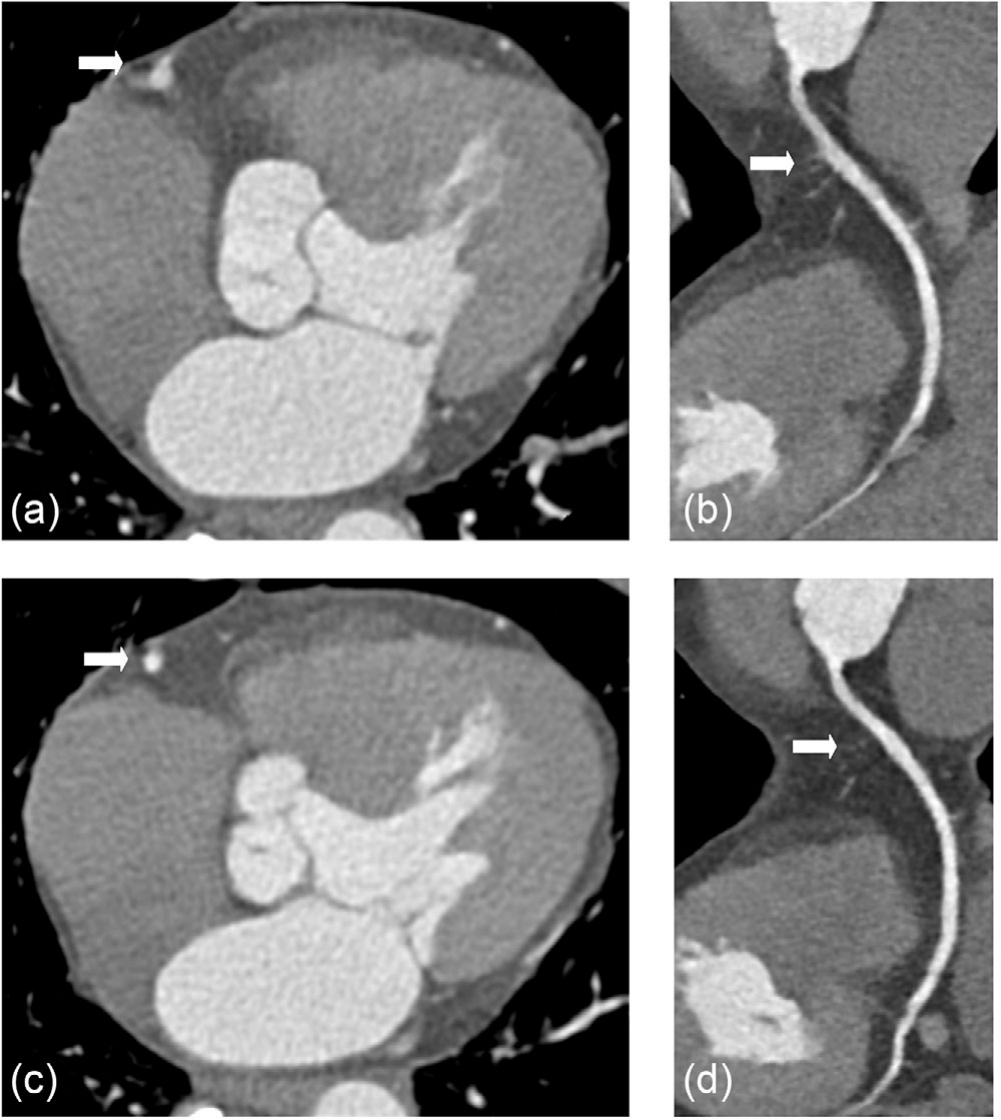
Example case of a 43‐year‐old male with an estimated heart rate of 78 bpm. On non‐ECG‐gated CCTA, images reconstructed with STD show a blurred RCA border with artefacts (arrows) on both the axial view (a) and curved planar reformation view (b). In contrast, after CMC, the corresponding axial (c) and curved planar reformation (d) images demonstrate a sharp RCA vessel border (arrows).

**FIGURE 6:**
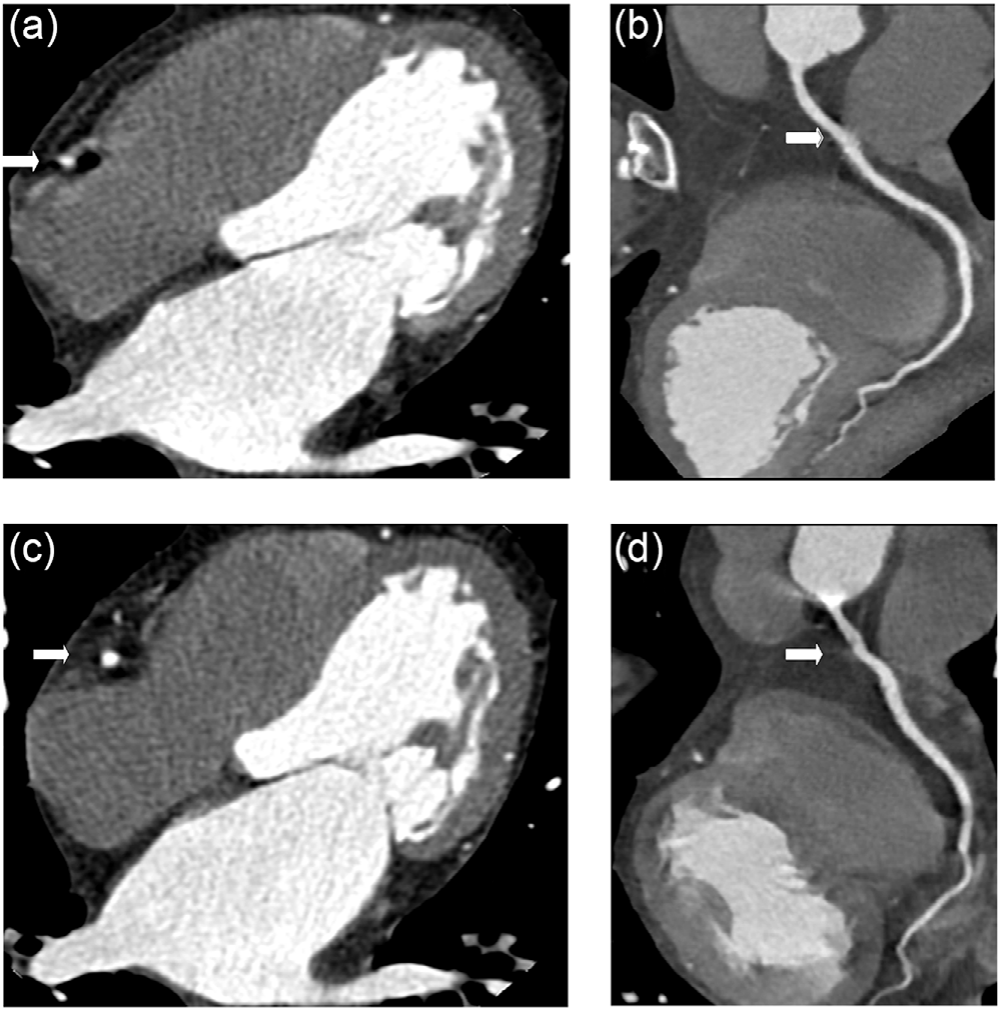
Example case of a 52‐year‐old female with an estimated heart rate of 81 bpm. On non‐ECG‐gated CCTA, images reconstructed with STD show a blurred RCA border with artefacts (arrows) on both the axial view (a) and curved planar reformation view (b). In contrast, after CMC, the corresponding axial (c) and curved planar reformation (d) images demonstrate a sharp RCA vessel border (arrows).

As shown in Table 4, no significant differences in SNR or CNR were observed between STD and CMC algorithms for any vessel or HR subgroups (all *p* > 0.05). In the high HR group, CMC significantly improved the vessel sharpness of the RCA compared with STD (*p* < 0.001). Figure 4 presents vessel sharpness measurements obtained with STD and CMC in non‐ECG‐gated CCTA at low, intermediate, and high HRs. Moreover, the CMC algorithm yielded a sharper, clearer vessel. Figure 5 and Figure 6 present representative axial and multiplanar reformation images from two patients reconstructed with STD and CMC, respectively.

**TABLE 4:** Objective image quality scores across different heart rates and algorithms.

	HR ≤ 60 bpm group (*n* = 27)			60 < HR ≤ 75 bpm group (*n* = 28)			HR > 75 bpm group (*n* = 21)		
	RCA	LAD	LCX	RCA	LAD	LCX	RCA	LAD	LCX
Signal‐to‐noise									
STD	25.37 ± 5.82	27.25 ± 4.16	27.38 ± 4.16	25.55 ± 5.57	26.60 ± 4.68	24.79 ± 4.74	24.30 ± 5.74	25.58 ± 4.67	21.76 ± 2.03
CMC	26.21 ± 4.53	28.07 ± 2.80	28.45 ± 5.47	26.58 ± 5.77	27.60 ± 4.86	26.04 ± 4.12	26.02 ± 5.30	25.66 ± 4.41	22.86 ± 4.96
*p*‐value	0.551	0.381	0.414	0.520	0.461	0.328	0.309	0.951	0.349
Contrast‐to‐noise									
STD	30.84 ± 5.71	33.65 ± 5.18	32.77 ± 7.48	29.65 ± 6.68	34.46 ± 4.34	30.90 ± 5.63	28.50 ± 7.36	29.36 ± 4.17	26.84 ± 5.56
CMC	32.64 ± 5.28	35.13 ± 4.71	33.45 ± 4.98	31.42 ± 6.09	35.36 ± 3.62	31.17 ± 5.25	29.87 ± 7.91	30.89 ± 5.08	27.67 ± 6.89
*p*‐value	0.223	0.264	0.729	0.326	0.432	0.861	0.558	0.281	0.665
Vessel sharpness									
STD CMC	219.37 ± 7.62	218.16 ± 4.63	226.27 ± 6.57	215.02 ± 14.44	211.76 ± 13.41	214.09 ± 7.86	183.44 ± 8.66	200.18 ± 11.28	210.99 ± 13.08
	223.42 ± 5.90	219.68 ± 4.56	228.91 ± 8.45	215.71 ± 11.44	212.08 ± 12.11	216.70 ± 5.92	204.39 ± 14.88	201.78 ± 7.16	214.74 ± 17.09
*p*‐value	0.703	1.000	1.000	1.000	1.000	1.000	**< 0.001** ^*^	1.000	1.000

*Note*: Data are presented as mean ± SD.

Abbreviation: AO, aortic root; CMC, cardiac motion correction; HR, heart rate; LAD, left anterior descending; LCX, left circumflex artery; RCA, right coronary artery; SNR, signal‐to‐noise ratio; CNR, contrast‐to‐noise ratio; STD, standard algorithm.

* *p*‐value was statistically significant.

## 4 DISCUSSION

In this study, we demonstrated the impact of CMC on image quality in CCTA across a range of HRs. The principal finding is that, compared with the STD, CMC plays a role in correcting coronary motion artifacts, resulting in significantly improved subjective image quality and vessel sharpness for the RCA at high (> 75 bpm) HRs. This suggests that CMC is a promising reconstruction algorithm for CCTA, with the potential to benefit a broader population of patients undergoing CCTA.

Although overall image quality was high in most subgroups, motion artifacts remained a challenge at HRs exceeding 75 bpm. Our study demonstrates that CMC could play a role in attenuating coronary motion artifacts. This finding is consistent with a previous study, demonstrating that motion‐compensated reconstruction (MCR) algorithms can significantly improve interpretability at the patient, vessel, and segment levels in patients with elevated heart rates (> 75 bpm).^24^ Notably, CMC improved the image quality of the RCA at high HRs. For instance, Liu et al.^19^ reported that the MCR algorithm reduced the proportion of non‐interpretable RCA segments from 39.2% to 3.8% (*p* < 0.001) at HRs ≥ 75 bpm. In this study, most non‐interpretable segments under high HR conditions were located in the proximal RCA. This may be attributable to the anatomical trajectory of the proximal RCA, which traverses the right atrioventricular groove in close proximity to the right atrium and right ventricle, thereby rendering it more susceptible to motion artifacts during cardiac systole.^25^

Compared with STD, CMC significantly improved the vessel sharpness of the RCA in CCTA under high HR conditions (STD: 183.44 ± 8.66, CMC: 204.39 ± 14.88, *p* < 0.001). By contrast, no statistically significant differences were observed between the two algorithms for the LAD or the LCX. These findings are consistent with prior investigations demonstrating a close relationship between the occurrence of coronary artery motion artifacts and the velocity of coronary artery movement. The RCA typically exhibits higher motion velocity than the LAD and LCX, rendering it more susceptible to motion artifacts during CCTA image acquisition and reconstruction.^26^ Achenbach et al.^27^ reported that the mean in‐plane motion velocity of the RCA was 69.5 ± 22.5 mm/s, compared with 22.4 ± 4.1 mm/s for the LAD and 48.4 ± 15.0 mm/s for the LCX, as measured by electron‐beam CT. As an effective motion compensation algorithm, CMC mitigates image blurring artifacts caused by rapid RCA motion by estimating and correcting the coronary motion vector field within the cardiac cycle.

In recent years, various techniques have been developed to mitigate coronary artery motion artifacts, primarily including hardware‐based strategies such as the application of 16‐cm wide‐detector technology. Compared with 6‐cm or 8‐cm detectors, the 16‐cm wide‐detector can cover the entire adult cardiac chamber (typically 120–140 mm) in a single acquisition.^11^ Furthermore, previous studies have demonstrated that higher temporal resolution is beneficial for improving CCTA image quality.^20^ The scanner used in this study has a gantry rotation speed of 0.259 s, providing corresponding temporal resolution. Yan et al.^28^ confirmed that rapid gantry rotation (0.25 s) combined with a wide‐detector array enables high‐quality coronary artery imaging within a single cardiac cycle, reducing inter‐slice registration errors and mitigating the effects of heart rate variability on image quality. In this study, CCTA image interpretability exceeded 90% at the per‐patient, per‐vessel, and per‐segment levels, which may be attributable to the aforementioned hardware characteristics providing robust support for motion correction algorithms to enhance image quality.

Despite the important findings of this study, several limitations should be acknowledged. First, this was a retrospective, single‐center study with a relatively limited sample size. Therefore, its findings may not be broadly generalizable and require validation in other populations and at other institutions. Second, although comparing diagnostic performance was not the primary aim of this study, we were unable to compare CCTA findings with invasive coronary angiography as a reference standard because these data were not available. Third, this study evaluated the impact of the CMC algorithm on image quality in non‐ECG‐gated CCTA across different heart rate conditions. Future research could explore its clinical utility in conventional ECG‐gated CCTA to more comprehensively assess its motion correction efficacy. Fourth, the diagnostic performance of CMC for assessing the degree of coronary stenosis or plaque characteristics was not evaluated. Future large‐scale, multicenter, prospective studies are needed to clarify this issue. Finally, the finger pulse oximeter used in this study cannot reliably detect arrhythmic events during CCTA. Consequently, the specific value of CMC in patients with cardiac arrhythmias warrants further investigation.

## 5 CONCLUSION

In conclusion, CMC is a promising reconstruction algorithm. Compared with STD, CMC reduced coronary motion blur, improved vessel delineation, and enhanced interpretability in non‐ECG‐gated CCTA, with significant differences observed in the image quality metrics of RCA at high (> 75 bpm) HRs.

## AUTHOR CONTRIBUTIONS

**MeiQi Wang**: Data curation; formal analysis; investigation; visualization; writing—original draft. **Li Tao**: data curation; formal analysis; conceptualization; methodology. **Wen Li**: resources; data curation; visualization; supervision. **YouHong Cao**: data curation; visualization; supervision. **Hao Wu**: data curation; visualization; supervision. **ZhiWei Zhang**: conceptualization; funding acquisition; investigation; methodology; resources; writing—review and editing. All authors read and approved the final manuscript. All authors read and approved the final manuscript.

## CONFLICTS OF INTEREST STATEMENT

All authors declare that they have no known competing financial interests or personal relationships that could have appeared to influence the work reported in this paper.

## ETHICAL APPROVAL

This study was approved by the Medical Research Ethics Review Committee of the First Affiliated Hospital of Chongqing Medical University (approval number: 2025‐298‐01).

## Data Availability

The datasets generated and/or analyzed during the current study are not publicly available due to hospital policy, but are available from the corresponding author on reasonable request.
